# Association of Serum Brain-Derived Tau With Clinical Outcome and Longitudinal Change in Patients With Severe Traumatic Brain Injury

**DOI:** 10.1001/jamanetworkopen.2023.21554

**Published:** 2023-07-03

**Authors:** Fernando Gonzalez-Ortiz, Maciej Dulewicz, Nicholas J. Ashton, Przemysław R. Kac, Henrik Zetterberg, Emma Andersson, Yara Yakoub, Jörg Hanrieder, Michael Turton, Peter Harrison, Bengt Nellgård, Thomas K. Karikari, Kaj Blennow

**Affiliations:** 1 Department of Psychiatry and Neurochemistry, Institute of Neuroscience and Physiology, the Sahlgrenska Academy at the University of Gothenburg, Gothenburg, Sweden; 2 Clinical Neurochemistry Laboratory, Sahlgrenska University Hospital, Mölndal, Sweden; 3 King’s College London, Institute of Psychiatry, Psychology and Neuroscience, Maurice Wohl Clinical Neuroscience Institute London, London, United Kingdom; 4 NIHR Biomedical Research Centre for Mental Health and Biomedical Research Unit for Dementia at South London and Maudsley NHS Foundation London, London, United Kingdom; 5 Centre for Age-Related Medicine, Stavanger University Hospital, Stavanger, Norway; 6 Department of Neurodegenerative Disease, UCL Institute of Neurology, Queen Square, London, United Kingdom; 7 UK Dementia Research Institute at UCL, London, United Kingdom; 8 Hong Kong Center for Neurodegenerative Diseases, Clear Water Bay, Hong Kong, China; 9 Wisconsin Alzheimer’s Disease Research Center, University of Wisconsin School of Medicine and Public Health, University of Wisconsin–Madison, Madison; 10 Department of Anaesthesiology and Intensive Care, Institute of Clinical Sciences, Sahlgrenska Academy, University of Gothenburg, Sweden; 11 Douglas Mental Health University Institute, Centre for Studies on the Prevention of Alzheimer’s Disease, Montreal, Quebec, Canada; 12 Bioventix, Surrey, United Kingdom; 13 Department of Anesthesiology, Surgery and Intensive Care; Sahlgrenska University Hospital, Mölndal, Sweden; 14 Department of Psychiatry, School of Medicine, University of Pittsburgh, Pittsburgh, Pennsylvania

## Abstract

**Importance:**

Blood-based measurements of total tau (T-tau) are commonly used to examine neuronal injury in patients with traumatic brain injury (TBI), but current assays do not differentiate between brain-derived tau (BD-tau) and tau produced in peripheral tissues. A novel assay for BD-tau has recently been reported that selectively quantifies nonphosphorylated tau of central nervous system origin in blood samples.

**Objectives:**

To examine the association of serum BD-tau with clinical outcomes in patients with severe TBI (sTBI) and its longitudinal changes over 1 year.

**Design, Setting, and Participants:**

This prospective cohort study was conducted at the neurointensive unit at the Sahlgrenska University Hospital, Gothenburg, Sweden, between September 1, 2006, and July 1, 2015. The study included 39 patients with sTBI followed up for up to 1 year. Statistical analysis was performed between October and November 2021.

**Exposures:**

Serum BD-tau, T-tau, phosphorylated tau_231_ (p-tau_231_), and neurofilament light chain (NfL) measured on days 0, 7, and 365 after injury.

**Main Outcomes and Measures:**

Associations of serum biomarkers with clinical outcome and longitudinal change in sTBI. Severity of sTBI was evaluated using the Glasgow Coma Scale at hospital admission, while clinical outcome was assessed with the Glasgow Outcome Scale (GOS) at 1-year follow-up. Participants were classified as having a favorable outcome (GOS score, 4-5) or unfavorable outcome (GOS score, 1-3).

**Results:**

Among the 39 patients (median age at admission, 36 years [IQR, 22-54 years]; 26 men [66.7%]) in the study on day 0, the mean (SD) serum BD-tau level was higher among patients with unfavorable outcomes vs those with favorable outcomes (191.4 [190.8] pg/mL vs 75.6 [60.3] pg/mL; mean difference, 115.9 pg/mL [95% CI, 25.7-206.1 pg/mL]), while the other markers had smaller between-group mean differences (serum T-tau, 60.3 pg/mL [95% CI, −22.0 to 142.7 pg/mL]; serum p-tau_231_, 8.3 pg/mL [95% CI, −6.4 to 23.0 pg/mL]; serum NfL, −5.4 pg/mL [95% CI, −99.0 to 88.3 pg/mL]). Similar results were recorded on day 7. Longitudinally, baseline serum BD-tau concentrations showed slower decreases in the whole cohort (42.2% on day 7 [from 138.6 to 80.1 pg/mL] and 93.0% on day 365 [from 138.6 to 9.7 pg/mL]) compared with serum T-tau (81.5% on day 7 [from 57.3 to 10.6 pg/mL] and 99.0% on day 365 [from 57.3 to 0.6 pg/mL]) and p-tau_231_ (92.5% on day 7 [from 20.1 to 1.5 pg/mL] and 95.0% on day 365 [from 20.1 to 1.0 pg/mL]). These results did not change when considering clinical outcome, where T-tau decreased twice as fast as BD-tau in both groups. Similar results were obtained for p-tau_231_. Furthermore, the biomarker levels on day 365 were lower, compared with day 7, for BD-tau but not T-tau or p-tau_231_. Serum NfL had a different trajectory to the tau biomarkers, with levels increasing by 255.9% on day 7 compared with day 0 (from 86.8 to 308.9 pg/mL) but decreasing by 97.0% by day 365 vs day 7 (from 308.9 to 9.2 pg/mL).

**Conclusions and Relevance:**

This study suggests that serum BD-tau, T-tau, and p-tau_231_ have differential associations with clinical outcome and 1-year longitudinal change in patients with sTBI. Serum BD-tau demonstrated utility as a biomarker to monitor outcomes in sTBI and can provide valuable information regarding acute neuronal damage.

## Key Points

**Question:**

Are levels of serum brain-derived tau (BD-tau) at admission associated with clinical outcome and long-term change in patients with severe traumatic brain injury (sTBI)?

**Findings:**

In this cohort study of 39 patients with sTBI, the mean fold difference in serum BD-tau concentrations on day 0 for patients with sTBI with unfavorable clinical outcomes vs those with favorable clinical outcomes 1 year after the injury was higher than the mean fold differences in serum total tau and phosphorylated tau_231_. Serum BD-tau demonstrated slower clearance from the blood (56.6% of baseline levels remaining by day 7) compared with total tau and phosphorylated tau_231_, which had only 19.0% and 7.5% of baseline levels, respectively, remaining at day 7.

**Meaning:**

This study suggests that concentrations and longitudinal trajectories of serum BD-tau differ among patients with sTBI depending on clinical outcome; serum BD-tau could be used as an accessible biomarker to monitor clinical outcome in patients with sTBI at admission and 7 days after the injury.

## Teaser

This cohort study examines the association of serum brain-derived tau with clinical outcomes and its longitudinal changes over 1 year among patients in Sweden with severe traumatic brain injury.

## Introduction

Traumatic brain injury (TBI) is one of the leading causes of morbidity, disability, and mortality across all ages.^1,2^ Around the world, more than 50 million individuals are affected by TBI every year.^2^ Posttraumatic complications of TBI can range from minor neurological and psychosocial problems to long-term disability,^3^ making it crucial to follow up with patients after injury to ascertain longitudinal outcomes.

Traumatic brain injury is often classified as mild or severe according to the intensity of the injury.^4^ Severe TBI (sTBI) can be more life threatening and has lower rates of survival.^2^ In clinical settings, sTBI is commonly classified using the Glasgow Coma Scale (GCS) at admission to the hospital, while the Glasgow Outcome Scale (GOS) is used to assess long-term clinical outcome.^5^ Moreover, structural damage after sTBI may be detected by neuroimaging techniques.^6^ Despite the proven effectiveness of these approaches, they have limitations in providing biochemical brain-related changes reflected in the bloodstream within a few hours after trauma. Circulating blood biomarkers provide biochemical information and prognostic insights into clinical severity to guide patient management and monitor long-term outcome.^4^

Serum total tau (T-tau) is one of the most well-characterized biomarkers for sTBI,^4,7,8^ showing high increases within hours of the injury.^7^ However, studies have suggested that current assays for T-tau quantify both central nervous system (CNS) and peripheral tau when measurements are performed on blood (serum or plasma) samples.^9,10^ Therefore, we hypothesized that a blood-based biomarker that is selective for CNS tau will be more accurate at reflecting the brain-associated tau released into the bloodstream while avoiding potential influences from peripheral tau. To this end, we evaluated the association of the novel brain-derived tau (BD-tau) marker^11^ with baseline clinical severity and longitudinal outcome compared with T-tau in serum samples from participants with sTBI followed up clinically over a 1-year period. We also examined changes in serum phosphorylated tau_231_ (p-tau_231_) and neurofilament light chain (NfL) concentrations as other neuronal injury-related markers.

## Methods

### Study Cohort, Design, and Outcome

This study included 42 participants (39 with data on all 4 serum biomarkers) from the prospective Swedish TBI Neurointensivvårdsavdelning cohort of patients with sTBI who were receiving clinical care at the Sahlgrenska University Hospital, Gothenburg, Sweden, and followed up for 1 year.^12,13^ Participant recruitment, clinical assessments, and blood sample collection were performed between September 1, 2006, and July 1, 2015. The inclusion criteria were (1) TBI with a GCS score of 8 or less on admission, (2) admission to the neurointensive care unit within 48 hours of head injury, (3) aged 18 years or older, (4) acceptance from next of kin to participate in the study, and (5) residence in Sweden for 12 months of follow-up. The exclusion criteria included no provision of informed consent, known history of neurological and/or autoimmune disease, and pregnancy. The ethics committee at the University of Gothenburg approved the study. Written informed consent was obtained from the patients’ proxies. This study followed the Strengthening the Reporting of Observational Studies in Epidemiology (STROBE) reporting guideline.

Traumatic brain injury outcome was clinically assessed with the GOS at 12 months^5^; those with a GOS score of 1 to 3 were classified as having an unfavorable outcome, and those with a GOS score of 4 to 5 were classified as having a favorable outcome. The 12-month outcome assessments were collected using a mixed-methods approach, including interviews performed either in person or via telephone. For participants with substantial impairment, their proxies were interviewed.

There were 39 participants on day 0, 39 on day 7, and 15 on day 365. Loss at follow-up was mainly due to death or disability, particularly in the group with unfavorable outcomes.

### Blood Sample Handling and Biomarker Measurements

Serum samples were obtained at the indicated time points according to standard procedures and stored at −80 °C until use. Serum BD-tau and p-tau_231_ were measured on the Simoa HD-X platform (Quanterix) using validated in-house assays,^11,14^ and T-tau and NfL with Quanterix assays (Nos. 101552 and 103670, respectively).

### Statistical Analysis

Biomarker measurements and statistical analyses were performed between October and November 2021 at the University of Gothenburg with Prism, version 9.3.1 (GraphPad). The distributions of data sets were examined for normality using the Kolmogorov-Smirnov test. Because the data were nonnormally distributed, nonparametric tests were used, and continuous data are presented as median (IQR) values. To compare serum biomarker levels between 2 groups (ie, unfavorable and favorable outcome groups at each time point), the mean fold differences (95% CI) were calculated and statistical comparisons examined using the Mann-Whitney test. For examining biomarker levels at all 3 time points (days 0, 7, and 365) within the whole cohort or the specific outcome groups, the Kruskal-Wallis test with the Dunn multiple comparison was used. *P* values (including those adjusted for multiple comparisons) were considered significant at the 2-sided *P* < .05 level.

## Results

### Cohort Characteristics

The study included 42 participants with at least 1 biomarker measured at baseline. However, 39 participants (median age at admission, 36 years [IQR, 22-54 years]; 26 men [66.7%]) had all measurements of BD-tau, T-tau, p-tau_231_, and NfL at baseline and were thus included in further analysis. There were no significant differences between the favorable and unfavorable outcome groups in age (median age, 36 years [IQR, 22-54 years] vs 31 years [IQR, 27-60 years]; *P* = .60) and sex (6 women and 12 men vs 7 women and 14 men; *P* = .07, determined by the Pearson χ^2^ test). The demographic characteristics of the cohort participants are summarized in the Table.

**Table.** Demographic Characteristics and Levels of Serum BD-Tau and Other Blood Biomarkers in sTBI

Characteristic	Unfavorable outcome	Favorable outcome	Mean difference (95% CI)
Sample size, No.	21	18	NA
Age at admittance, mean (SD), y	34.2 (15.5)	35.1 (17.5)	NA
Sex, No. (%)			
Female	7 (33.3)	6 (33.3)	NA
Male	14 (66.7)	12 (66.7)	NA
**Day 0**			
Sample size, No.	21	18	NA
Sex, No. (%)			
Female	7 (33.3)	6 (33.3)	NA
Male	14 (66.7)	12 (66.7)	NA
Serum BD-tau, mean (SD), pg/mL	191.4 (190.8)	75.6 (60.3)	115.9 (25.7 to 206.1)
Serum total tau, mean (SD), pg/mL	86.7 (177.0)	26.3 (40.1)	60.3 (−22.0 to 142.7)
Serum p-tau_231_, mean (SD), pg/mL	22.5 (29.1)	14.2 (14.1)	8.3 (−6.4 to 23.0)
Serum NfL, mean (SD), pg/mL	85.7 (66.7)	91.1 (186.8)	−5.4 (−99.04 to 88.3)
**Day 7**			
Sample size, No.	21	18	NA
Sex, No. (%)			
Female	7 (33.3)	6 (33.3)	NA
Male	14 (66.7)	12 (66.7)	NA
Serum BD-tau, mean (SD), pg/mL	124.2 (167.6)	44 (31.1)	80.2 (4.7 to 155.8)
Serum total tau, mean (SD), pg/mL	18.6 (42.4)	2.7 (3.3)	15.9 (−3.4 to 35.3)
Serum p-tau_231_, mean (SD), pg/mL	1.7 (1.5)	1.5 (1.1)	0.2 (−0.7 to 1.0)
Serum NfL, mean (SD), pg/mL	380.5 (305.1)	233 (174.1)	147.2 (−5.9 to 300.4)
**Day 365**			
Sample size, No.	4	11	NA
Sex, No. (%)			
Female	1 (25.0)	4 (36.4)	NA
Male	3 (75.0)	7 (63.6)	NA
Serum BD-tau, mean (SD), pg/mL	3.1 (0.3)	12.9 (20.3)	−9.9 (−23.5 to 3.8)
Serum total tau, mean (SD), pg/mL	0.47 (0.7)	0.6 (0.4)	−0.2 (−1.2 to 0.9)
Serum p-tau_231_, mean (SD), pg/mL	0.7 (0.6)	1.1 (0.5)	−0.4 (−1.2 to 0.3)
Serum NfL, mean (SD), pg/mL	20.7 (42.8)	3.9 (1.8)	16.8 (−36.2 to 69.8)

Abbreviations: BD-tau, brain-derived tau; NA, not applicable; NfL, neurofilament light chain; p-tau_231_, phosphorylated tau_231_; sTBI, severe traumatic brain injury.

### Serum BD-Tau Levels in sTBI Clinical Outcome Groups on Admission and 7 Days Later

Initial levels of BD-tau (on days 0 and 7) were associated with GOS outcome at 1 year. Thus, on day 0, mean (SD) serum BD-tau levels were higher in the unfavorable outcome group (191.4 [190.8] pg/mL) vs the favorable outcome group (75.6 [60.3] pg/mL; mean difference, 115.9 pg/mL [95% CI, 25.7-206.1 pg/mL]). However, the other markers had smaller between-group mean differences (serum T-tau, 60.3 pg/mL [95% CI, −22.0 to 142.7 pg/mL]; serum p-tau_231_, 8.3 pg/mL [95% CI, −6.4 to 23.0 pg/mL]; serum NfL, −5.4 pg/mL [95% CI, −99.0 to 88.3 pg/mL]) (Table). Similar results were recorded on day 7.

### Serum BD-Tau Longitudinal Trajectory vs Other Biomarkers

In the whole cohort, baseline serum BD-tau levels decreased 42.2% by day 7 (from 138.6 to 80.1 pg/mL) and 93.0% by day 365 (from 138.6 to 9.7 pg/mL) (Figure). When comparing serum BD-tau level on day 7 with clinical outcome at 1 year in both outcome groups, we found a smaller decrease in BD-tau level in the unfavorable (by day 7: 35.1% [from 191.4 to 124.2 pg/mL]; by day 365: 97.0% [from 124.2 to 3.1 pg/mL]) vs favorable (by day 7: 41.8% [from 75.6 to 44.0 pg/mL]; by day 365: 70.5% [from 44.0 to 13.0 pg/mL]) outcome group (Table). However, despite the decrease in concentrations, the mean differences between outcome groups were similar at days 0 and 7. By day 365, serum BD-tau levels in both groups had further decreased to concentrations that were much lower than the corresponding day 0 and day 7 levels (Figure, A).

**Figure.**
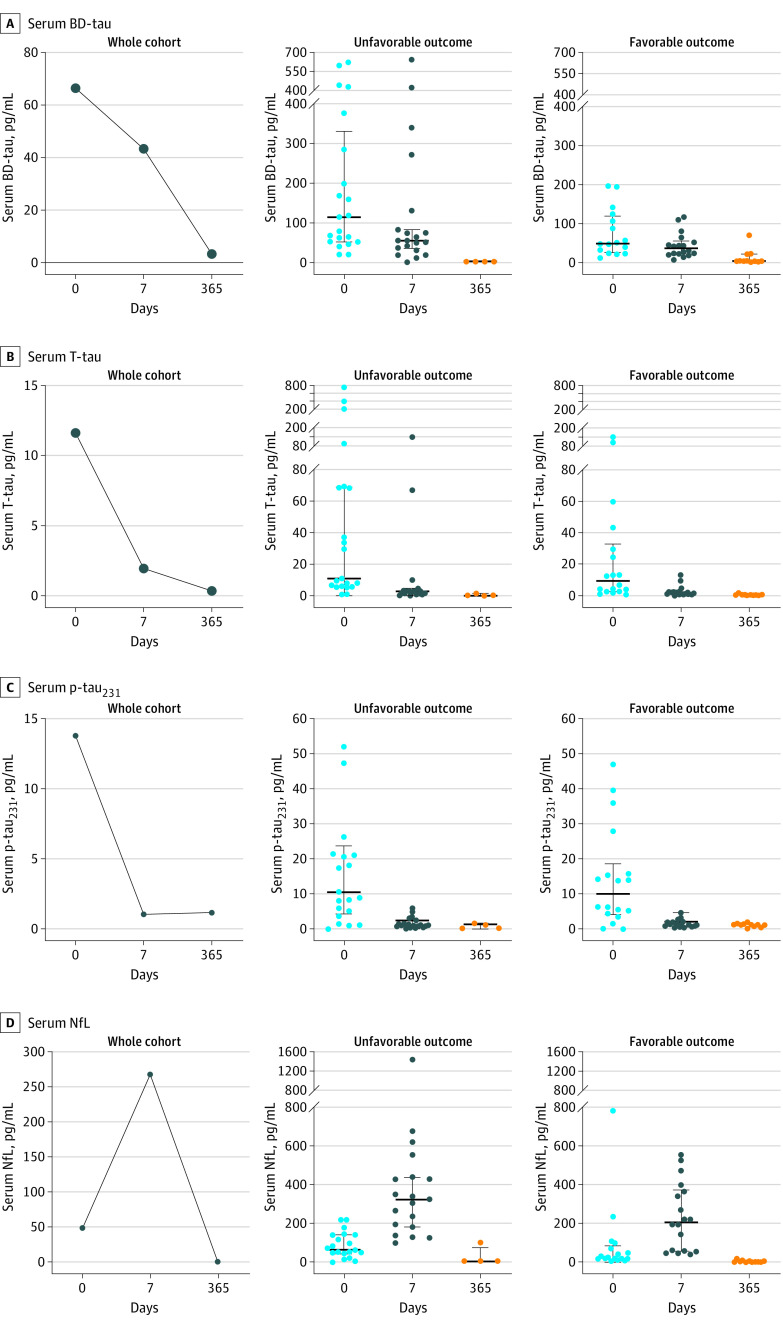
Longitudinal Trajectories of Serum Biomarker Levels After Severe Traumatic Brain Injury (TBI), in the Whole Cohort and According to Clinical Outcome Longitudinal trajectories of serum biomarker levels following severe TBI, in the whole cohort and according to clinical outcome. The plots show the median serum concentrations of brain-derived tau (BD-tau) (A), total tau (T-tau) (B), phosphorylated tau_231_ (p-tau_231_) (C), and neurofilament light chain (NfL) (D). In each plot, the serum biomarker values at different time points (on days 0, 7, and 365) are shown for the whole cohort (left) as well as in the 2 clinical outcome groups. The time-dependent biomarker dynamics plot for the unfavorable outcome group is shown in the middle and that of the favorable outcome group is plotted to the right. Note that serum BD-tau levels are higher in absolute values (ie, picograms per milliliter) than T-tau not because BD-tau picks larger amounts of tau in serum. However, this observation is due to the use of different assay designs, calibrators, and standard curves for each biomarker, which means that these values are not directly comparable in the numerical sense. Error bar indicates IQR.

Serum T-tau and p-tau_231_ levels were also decreased between days 0 and 7 (Table and Figure, B and C). However, the decreases were larger compared with BD-tau levels, both for the whole cohort (day 7: 81.5% for T-tau [from 57.3 to 10.6 pg/mL] and 92.5% for p-tau_231_ [from 20.1 to 1.5 pg/mL]; day 365: 99.0% for T-tau [from 10.6 to 0.6 pg/mL] and 95.0% for p-tau_231_ [from 20.1 to 1.0 pg/mL]) as well as according to clinical outcome (T-tau: 89.7% for favorable outcome [from 26.4 to 2.7 pg/mL] and 78.5% for unfavorable outcome [from 86.7 to 18.6 pg/mL]; p-tau_231_: 89.4% for favorable outcome [from 14.2 to 1.5 pg/mL] and 92.4% for unfavorable outcome [from 22.5 to 1.7 pg/mL]; Figure, B and C). Another distinction from BD-tau levels was that the mean differences between groups on days 0 and 7 tended to vary (Table). Because of the decrease in concentration for T-tau and p-tau_231_ from day 0 to day 7, the between-group mean differences on day 7 vs day 365 were similar.

The longitudinal trajectory of serum NfL was different from the longitudinal trajectories of the tau-based biomarkers. Instead of decreasing from day 0 to day 7, serum NfL increased by 255.5% (from 86.8 to 308.9 pg/mL). There were increases of 156.0% (from 91.1 to 233.2 pg/mL) and 343.5% (from 85.8 to 380.5 pg/mL) from day 0 in the favorable and unfavorable outcome groups, respectively (Table and Figure). The highest levels were recorded on day 7 in the whole cohort and in both clinical outcome groups, with increased mean differences between days 0 and 7 (Table). The levels decreased by 97.0% (from 308.9 to 9.2 pg/mL) from day 7 to day 365 (Table and Figure, D).

## Discussion

In the present study, we found that serum BD-tau level could have utility for evaluating clinical outcome in sTBI, both on the day of the event and 7 days later. These results, which were not observed for serum T-tau level, suggest that the selective measurement of tau of CNS origin in the bloodstream has the capacity to improve the accuracy of the clinical outcome and management of sTBI. In agreement with recent findings indicating that current blood-based T-tau assays quantify tau of both CNS and peripheral origin and that the latter makes up approximately 80% of T-tau signal in the bloodstream,^15^ our results suggest that CNS tau differences in groups of patients with sTBI and in different clinical outcomes can be masked if a nonselective blood-based tau assay (ie, T-tau) is used. In addition, the inability of p-tau_231_ and NfL to differentiate between the clinical outcome groups suggests their limited value for the clinical evaluation of sTBI, despite their well-validated functions for Alzheimer disease pathophysiology and general neurodegeneration, respectively.^14,16^

The results indicate that all 3 tau biomarkers (BD-tau, T-tau, and p-tau_231_) are released from the brain into the bloodstream within minutes to hours of sTBI, possibly due to the opening of the blood-brain barrier. This initial increased release of both total (unphosphorylated) and phosphorylated forms of tau agrees with previous reports showing that brain trauma leads to the rapid release of tau of various molecular forms into extracellular fluids.^7,8^ The consistent longitudinal reduction in these biomarker levels was due to a lack of replenishment of the initial (day 0) signals during physiologically regulated tau turnover.^7^ Serum T-tau was cleared much more rapidly (81% removed by day 7) than BD-tau, which could be explained by the ratio of CNS tau to peripheral tau in the bloodstream, which was in favor of CNS tau on day 0 (due to increased release of brain tau) returning to pre-sTBI levels over time. However, BD-tau, which exclusively quantifies brain-originating tau,^11^ showed that CNS tau is not cleared so quickly and that substantial amounts do remain for up to 1 year. This slow clearance of BD-tau proved useful for the clinical monitoring of outcome and recovery after sTBI. For example, while considering T-tau alone might suggest recovery by day 7 (due to significantly decreased levels of BD-tau compared with day 0), BD-tau indicates otherwise because the levels were still statistically indifferent from day 0 regardless of clinical outcome. Continuous evaluation of BD-tau levels between days 7 and 365 would be informative to ascertain the point at which the decrease was significantly lower compared with days 0 and 7 and whether patients with favorable outcomes reached this point earlier than those with unfavorable outcomes. We also anticipate that individuals with mild TBI will show faster decreases in BD-tau compared with those with sTBI. Finally, NfL had a different trajectory, similar to previous reports,^12^ suggesting slower release into the bloodstream compared with the tau markers. However, the peaking of the signal at day 7 and its difference from day 365 suggests its value for outcome monitoring after sTBI.

### Strengths and Limitations

This study has some strengths, including the longitudinal design and that multiple biomarkers were compared head to head. It also has some limitations, including the lack of sampling time points between days 7 and 365, as well as the restriction of the study to patients with sTBI without including those with mild TBI. Moreover, many participants were lost at follow-up due to death and disability. Despite the important and novel information provided in this study, the results should be independently validated in cohorts with larger sample sizes across time points. In addition, control groups of uninjured individuals as well as those with orthopedic injuries were lacking. Data were collected using GOS and not the Glasgow Outcome Sclae–Extended as the main outcome score. The limited subcharacterization of the sTBI outcome groups (eg, using functional and neuroimaging measures) limited further stratification of the participants with extremely high and low biomarker levels in both the unfavorable and favorable outcome groups.

## Conclusions

In this cohort study, serum BD-tau level showed the capacity to differentiate clinical outcomes on the day of sTBI and 7 days later and to follow the dynamics of CNS-derived tau over 1 year. The longitudinal changes in BD-tau level did differ from the level of T-tau and p-tau_231_, which decreased much faster (most signals had been removed by day 7), and NfL, which showed a slower pattern of release into the bloodstream. These findings support the value of serum BD-tau level as a biomarker to monitor outcomes in patients with sTBI.

## Notes

Supplement.
Data Sharing Statement
