# Supplementary material for: Association of Serum Brain-Derived Tau With Clinical Outcome and Longitudinal Change in Patients With Severe Traumatic Brain Injury
Source: JAMA Netw Open. 2023 Jul 3;6(7):e2321554. doi: 10.1001/jamanetworkopen.2023.21554 (PMC10318474; doi:10.1001/jamanetworkopen.2023.21554)
Supplement: Supplement. — Data Sharing Statement [file jamanetwopen-e2321554-s001.pdf]

Data Sharing Statement

Gonzalez-Ortiz. Association of Serum Brain-Derived Tau With Clinical Outcome and Longitudinal Change in Patients With Severe Traumatic Brain Injury. *JAMA Netw Open*. Published July 03, 2023. doi:10.1001/jamanetworkopen.2023.21554

Data
Data available: No

Additional Information
Explanation for why data not available: Patients data is protected
